# Influenza D virus M2 protein exhibits ion channel activity in *Xenopus laevis* oocytes

**DOI:** 10.1371/journal.pone.0199227

**Published:** 2018-06-21

**Authors:** Evan Kesinger, Jianing Liu, Aaron Jensen, Catherine P. Chia, Andrew Demers, Hideaki Moriyama

**Affiliations:** School of Biological Sciences, University of Nebraska-Lincoln, Lincoln, Nebraska, United States of America; Institut de Genetique et Developpement de Rennes, FRANCE

## Abstract

**Background:**

A new type of influenza virus, known as type D, has recently been identified in cattle and pigs. Influenza D virus infection in cattle is typically asymptomatic; however, its infection in swine can result in clinical disease. Swine can also be infected with all other types of influenza viruses, namely A, B, and C. Consequently, swine can serve as a “mixing vessel” for highly pathogenic influenza viruses, including those with zoonotic potential. Currently, the only antiviral drug available targets influenza M2 protein ion channel is not completely effective. Thus, it is necessary to develop an M2 ion channel blocker capable of suppressing the induction of resistance to the genetic shift. To provide a basis for developing novel ion channel-blocking compounds, we investigated the properties of influenza D virus M2 protein (DM2) as a drug target.

**Results:**

To test the ion channel activity of DM2, the DNA corresponding to DM2 with cMyc-tag conjugated to its carboxyl end was cloned into the shuttle vector pNCB1. The mRNA of the DM2–cMyc gene was synthesized and injected into *Xenopus* oocytes. The translation products of DM2–cMyc mRNA were confirmed by immunofluorescence and mass spectrometry analyses. The DM2–cMyc mRNA-injected oocytes were subjected to the two-electrode voltage-clamp (TEVC) method, and the induced inward current was observed. The midpoint (V_mid_) values in Boltzmann modeling for oocytes injected with DM2–cMyc RNA or a buffer were −152 and −200 mV, respectively. Assuming the same expression level in the *Xenopus* oocytes, DM2 without tag and influenza C virus M2 protein (CM2) were subjected to the TEVC method. DM2 exhibited ion channel activity under the condition that CM2 ion channel activity was reproduced. The gating voltages represented by V_mid_ for CM2 and DM2 were –141 and –146 mV, respectively. The reversal potentials observed in ND96 for CM2 and DM2 were −21 and −22 mV, respectively. Compared with intact DM2, DM2 variants with mutation in the YxxxK motif, namely Y72A and K76A DM2, showed lower V_mid_ values while showing no change in reversal potential.

**Conclusion:**

The M2 protein from newly isolated influenza D virus showed ion channel activity similar to that of CM2. The gating voltage was shown to be affected by the YxxxK motif and by the hydrophobicity and bulkiness of the carboxyl end of the molecule.

## Introduction

Influenza virus can infect various animal species, including humans [1, 2]. Although most influenza virus infections result in mild disease, genetic shift, drift, and reassortment events have been shown to result in highly pathogenic strains [3]. To date, four influenza virus species have been identified, namely A, B, C, and D [2, 4]. Type A infects several species, including humans as well as porcine, bovine, and canine species [5]. Types B and C infect humans and pigs [6]. Type D is a relatively newly identified type of influenza virus, which has been found to infect cattle and pigs [4]; it was recognized as a new virus type by the International Committee of Taxonomy in 2016 (talk.ictvonline.org).

Influenza D virus infection in cattle is typically asymptomatic [7, 8]. However, its infection in swine can result in clinical disease. Swine can also be infected with all other types of influenza viruses (types A—C). Consequently, swine can serve as a “mixing vessel” for highly pathogenic influenza viruses, including those with zoonotic potential [9]. A recent study has confirmed that people working in close proximity to calves have higher rates of seropositivity to the influenza virus (94%) than the general population (1.3%) [10].

**Fig 1:**
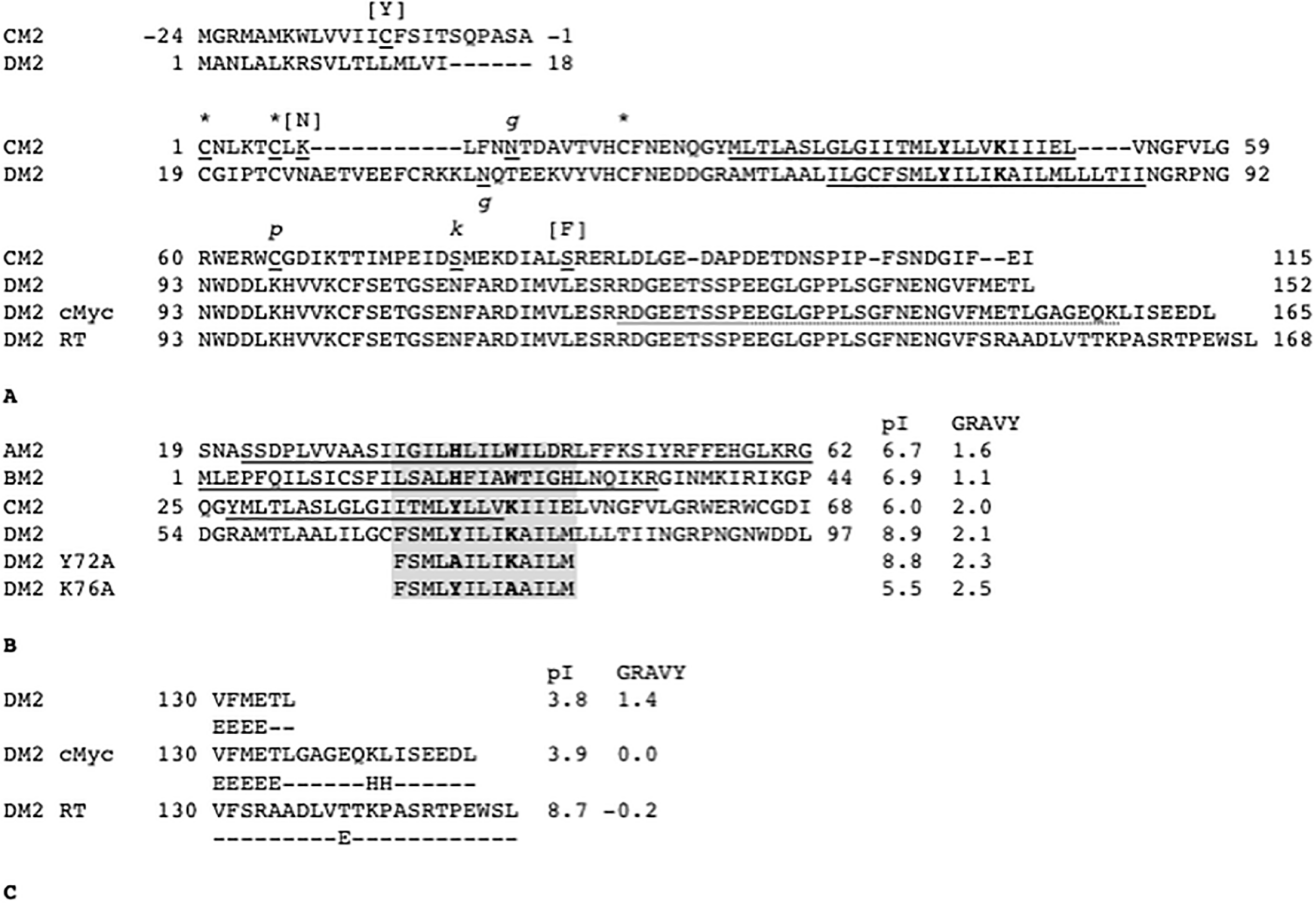
Amino acid sequences. A. Primary structures of influenza C virus M2 protein (CM2; Sequence ID, YP_089658.1 (C/Ann Arbor/1/1950)) and influenza D virus M2 protein (DM2; AFJ19025 (D/Swine/Oklahoma/1334/2011)). CM2 contains a 24-amino acid signal sequence (−24 to −1). Mature CM2 starts with Cys1 and contains extracellular, transmembrane (underlined), and internal domains in order from the amino to carboxyl terminals. Amino acid substitutions between Yamagata [BAA03793.1 (C/Yamagata/1/1988)] and Ann Arbor isolates are indicated in brackets. CM2 has disulfide-linked oligomerization sites (“*”; Cys1, Cys6, Cys20), a glycosylation site (Asn11, “*g*”), and a palmitoylation site (Cys65, “*p*”). A phosphorylation site involved in efficient virus replication is also indicated (Ser68, “*k*”) [11]. In DM2, the predicted transmembrane domain is indicated by an underline. The YxxxK motif, Tyr72, and Lys76 in D is indicated by bold face. DM2 has a potential glycosylation site at Asn39 indicated by “*g*”. A C-terminal variant in D cMyc has a spacer GAG and a cMyc-tag EQKLISEEDL. A readthrough C-terminal variant of DM2, DM2 RT, has an extra peptide from the vector pNCB1. B. Structures of M2 proteins. AM2. The NMR structure (residues 22–62; PDB ID, 2L0J) and crystallographic structure (21–46; 4QKL) of influenza A virus M2 protein [AAA43303 [A/Udorn/1972(H3N2)]]. BM2. The NMR structure (1–33; 2KIX) of influenza B virus M2 protein [ACF54325.1 (B/Taiwan/70061/2006)]. CM2. The structure of influenza C virus M2 protein (27–46) was obtained by site-specific infrared dichroism [12]. DM2. Influenza D virus M2 protein is focused on in this study. Solved or predicted structures are underlined. Amino acid residues providing experimental structure information are indicated by underlines. Primary structures of mutated DM2 are added with the isoelectric point (pI) and the hydrophobicity value (GRAVY) for the shaded helix domain. In GRAVY, greater positive values indicate higher hydrophobicity. C. Predicted secondary structure of carboxyl ends in DM2 constructs. Predicted secondary structures E and H correspond to β-strand and α-helix, respectively.

Administration of an efficacious vaccine is most effective in prevention of Influenza infection achieved [13, 14]. However, if infection has already occurred, the only treatment option is the use of antivirals. Unfortunately, owing to the inherent instability and high mutation rate of influenza genomes, most current antivirals are no longer effective at inhibiting influenza virus replication. The only currently available antiviral that targets the influenza AM2 protein ion channel (M2; Fig 1) [15, 16], called amantadine (PubChem CID, 2130), is only partially effective. Thus, there is an urgent need to develop an M2 ion channel blocker capable of suppressing the induction of resistance.

The influenza A virus M2 protein (AM2) is involved in the release of viral RNPs from the endosome (uncoating) and the transport of hemagglutinin to the cell surface via the trans-Golgi network [17–19]. In both processes, acidification of the external environment around the virus activates the M2 proton channel capability, leading to virus disassembly.

**Fig 2:**
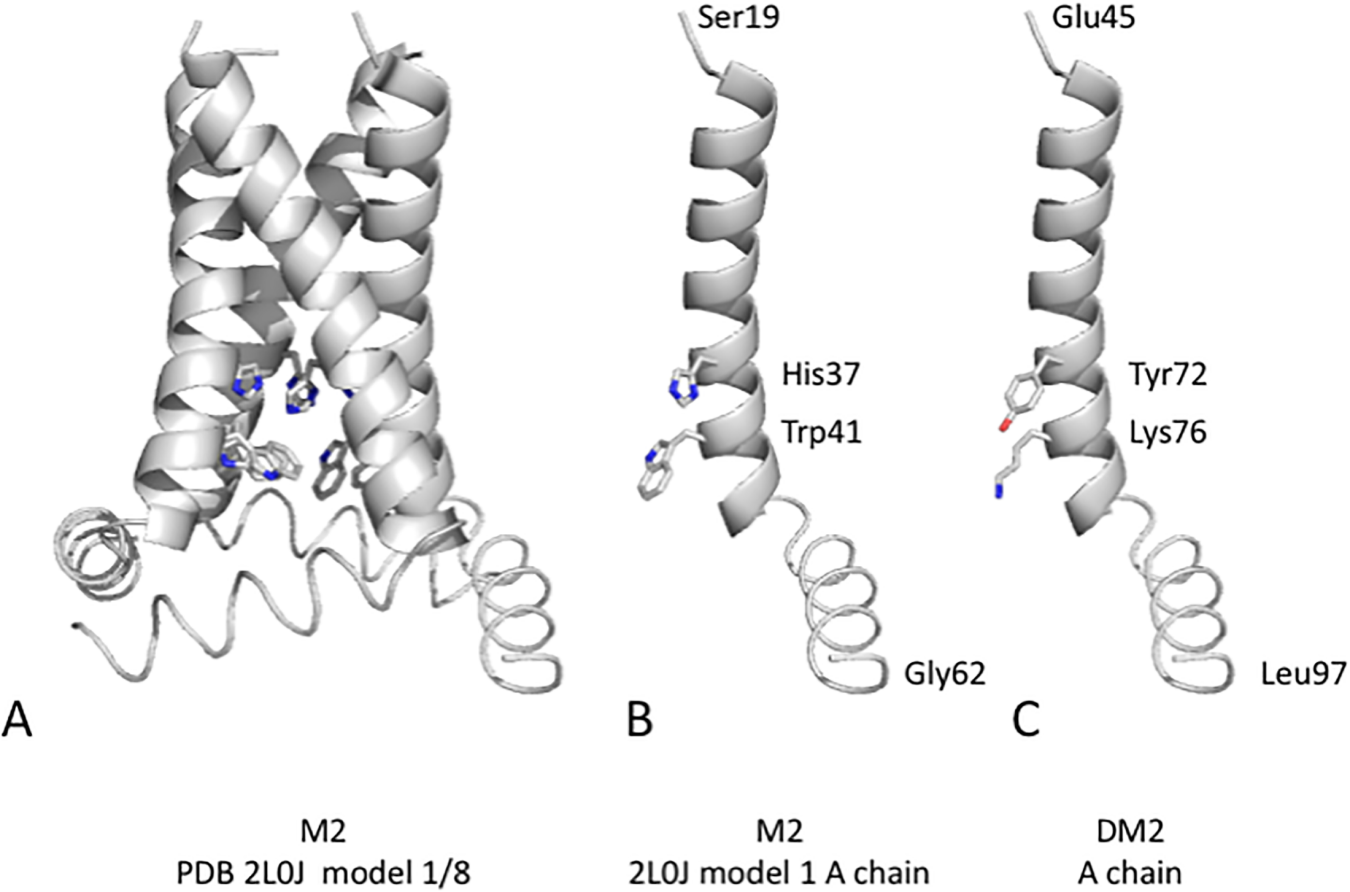
3D structure of M2 protein. A. The structure of AM2 was solved experimentally by nuclear magnetic resonance spectroscopy (NMR; structures were deposited in the Protein Data Bank with ID code 2L0J; model 1 is shown while the NMR structure contains 8 isomers). AM2 adopts a homo-tetramer configuration, and the middle of the assembly contains a pore. The valve residues His37 and Trp41 face inward within the pore on each monomer. B. The monomer structure of AM2 with valve residues, which control ion flow by pseudo-cation—pi interaction. C. A theoretical model of DM2 in monomer format.

The crystal structure of AM2 transmembrane helix has been solved at high resolution (PDB ID, 4QKL, deposited in monomer format) [20]. Based on this model, electrophysiological behaviors of the pH-gated M2 proton channel were explained by simulations [21]. Specifically, Val27 and Ser31 in the amino terminal and His37 and Trp41 in the carboxyl terminal were shown to act as a hinge and a gate, respectively (referred to as the HxxxW motif hereafter) [22]. Solid state NMR structures of the channel domain of M2 (2L0J; deposited in tetramer format; Fig 2) [23] were also reported.

The influenza B virus M2 protein (BM2) also contains the HxxxW motif [22], and the protein structure in micelle was solved by solution state NMR (2KIX; deposited in tetramer format) [24].

The influenza C virus M2 protein (CM2) was reported by Hongo *et al*. [25] [(C/Yamagata/1/1988); Fig 1], and it was identified to function as a voltage activated chloride ion channel [26]. Although CM2 does not contain the HxxxW motif, the helical region contains a YxxxK motif, as revealed by our visual observations (Figs 1 and 2). CM2 undergoes a proteolytic naturalization process that removes the leading 24 amino acids [27]. Mature CM2 comprises an amino-terminal extracellular domain, transmembrane domain, and a carboxyl-terminal internal domain [27, 28]. In a different isolate, (C/Ann Arbor/1/1950) [29], a transmembrane domain (Tyr27–Val46) was predicted by site-specific infrared dichroism analysis and a global molecular dynamics search [12]. Side chains of L31, L34, M41, and L44 residues were predicted to point to the center of the tetramer assembly. CM2 undergoes oligomerization by disulfide bonding among Cys1–Cys6–Cys20 [30]. CM2 undergoes glycosylation at Asn11 [27, 28] and palmitoylation at Cys65 [31]. Three amino acid substitutions that differ between Yamagata and Ann Arbor isolates were found to be located outside the transmembrane domains.

The type D proteome (D/Swine/Oklahoma/1334/2011), including the M2 protein, shares more sequence similarity with type C proteome than with type A or B proteome [7]. As such, many of our inferences about the structure and function of the DM2 protein are derived from what we know about CM2. We hypothesized that DM2 has voltage-gated chloride ion channel activity similar to that of CM2 and that this channel might depend on the YxxxK motif present in both channels (Figs 1 and 2c). To test the hypothesis, we used the two-electrode voltage-clamp (TEVC) technique coupled with a heterologous expression system with *Xenopus* oocytes because this system is well established.

## Results

### Expression of DM2 protein in *Xenopus* oocytes

**Fig 3:**
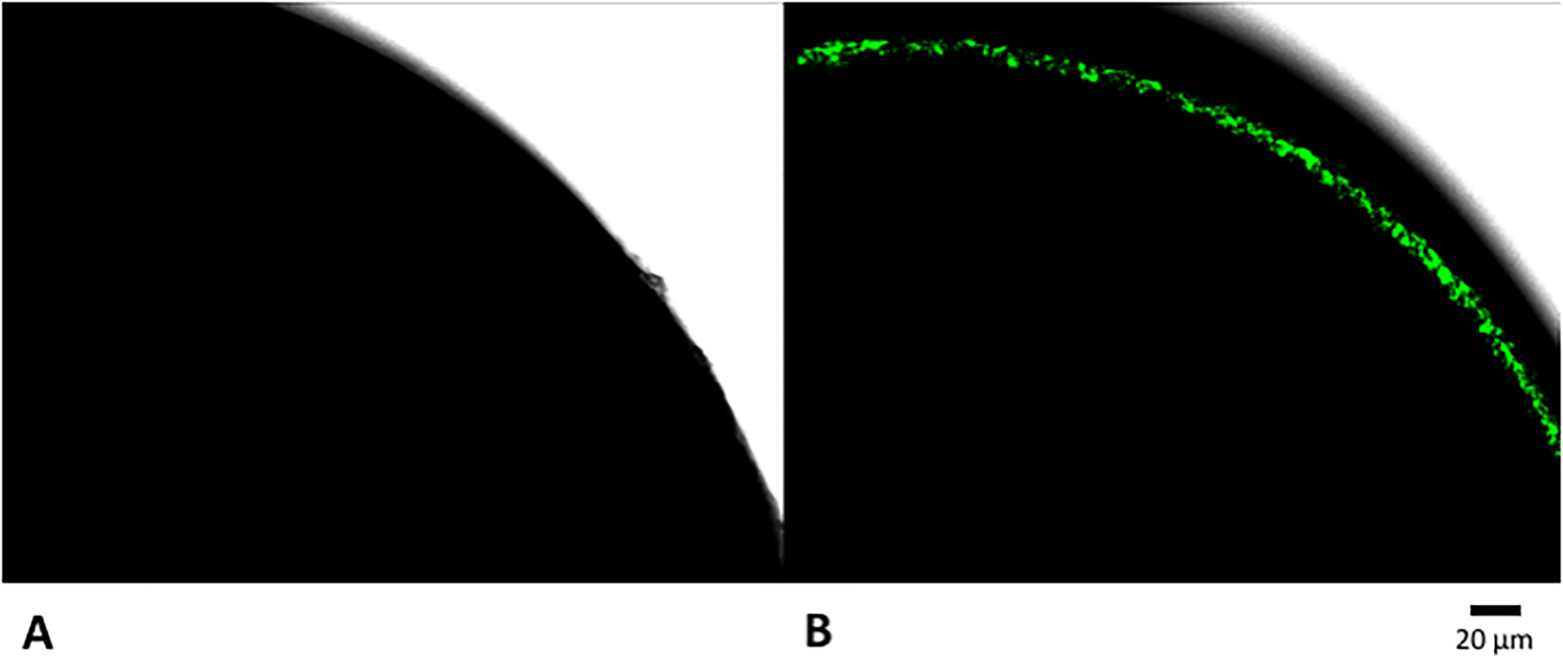
Confocal microscopy of oocytes. A. An oocyte injected with phosphate-buffered saline. B. An oocyte injected with RNA encoding c-Myc-tagged DM2. Oocytes were treated with anti-cMyc antibodies (mouse 9E10) and then donkey anti-mouse Alexa Fluor 488 antibodies.

A DM2 construct with a cMyc-tag at its carboxyl end was used to express DM2 in a *X*. *laevis* oocyte (Fig 1). The cMyc-tagged DM2 was expressed under the *Xenopus* β-globin 5′-UTR in the pNCB1 vector, which was adapted from pGEMHE [32]. *In vitro* transcribed mRNA was injected into the oocyte, followed by incubation in ND96 medium at 18°C for 5 days to maximize protein accumulation. An oocyte injected with phosphate-buffered saline (PBA), followed by incubation in ND96 medium at 18°C for 5 days, was considered as the control. The injected oocytes were stained to detect the DM2 protein through a cMyc epitope-tag using primary 9E10 antibody and secondary Alexa Fluor 488 antibody. The stained oocytes were observed by confocal microscopy (Fig 3). A concentric green layer was observed in the oocyte injected with RNA encoding cMyc-tagged DM2, whereas the control oocyte showed no fluorescence. This result seemed to be consistent with the observation of CM2 by Hongo *et al*. [25], although they used rabbit immune serum against a GST fusion protein containing CM2. Our results indicated that cMyc-tagged DM2 was produced in the *Xenopus* oocyte upon the injection of its RNA and formed a layer near the oocyte surface.

**Fig 4:**
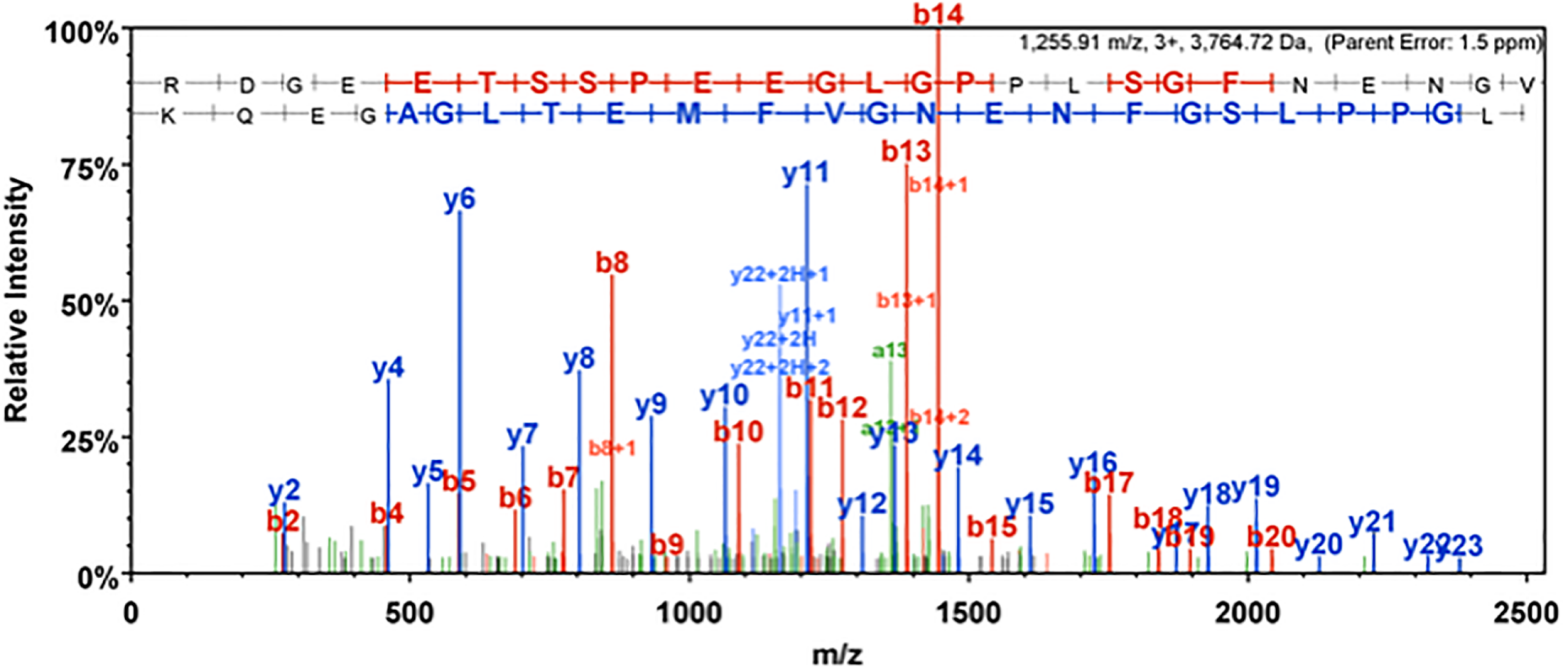
Mass spectrometry and peptide identification. Presented the MS/MS fragmentation spectrum of the 36 amino acids peptide identified, Arg123 –Lys158. The oocyte injected with RNA encoding DM2–cMyc was subjected to tryptic digestion followed by mass spectrometry using a nano-LC-MS/MS set-up. At 1255.91, a peptide of 36-amino acid long which includes the C-terminal region, the linker, and a portion of the cMyc sequence (Fig 1) was confidently identified with m/z value. The 20 and 17 successive peptide N-terminus retaining Y (blue) and peptide N-terminus retaining B fragment ions (red) matched the peptide sequence with a parent ion mass error of 1.5 ppm. The identified amino acid sequence for Y peptide shown is backward (blue). The S1 Table shows the fragmentation table for the corresponding peptide Arg123 –Lys158 representing both b- and y-ions.

**Fig 5:**
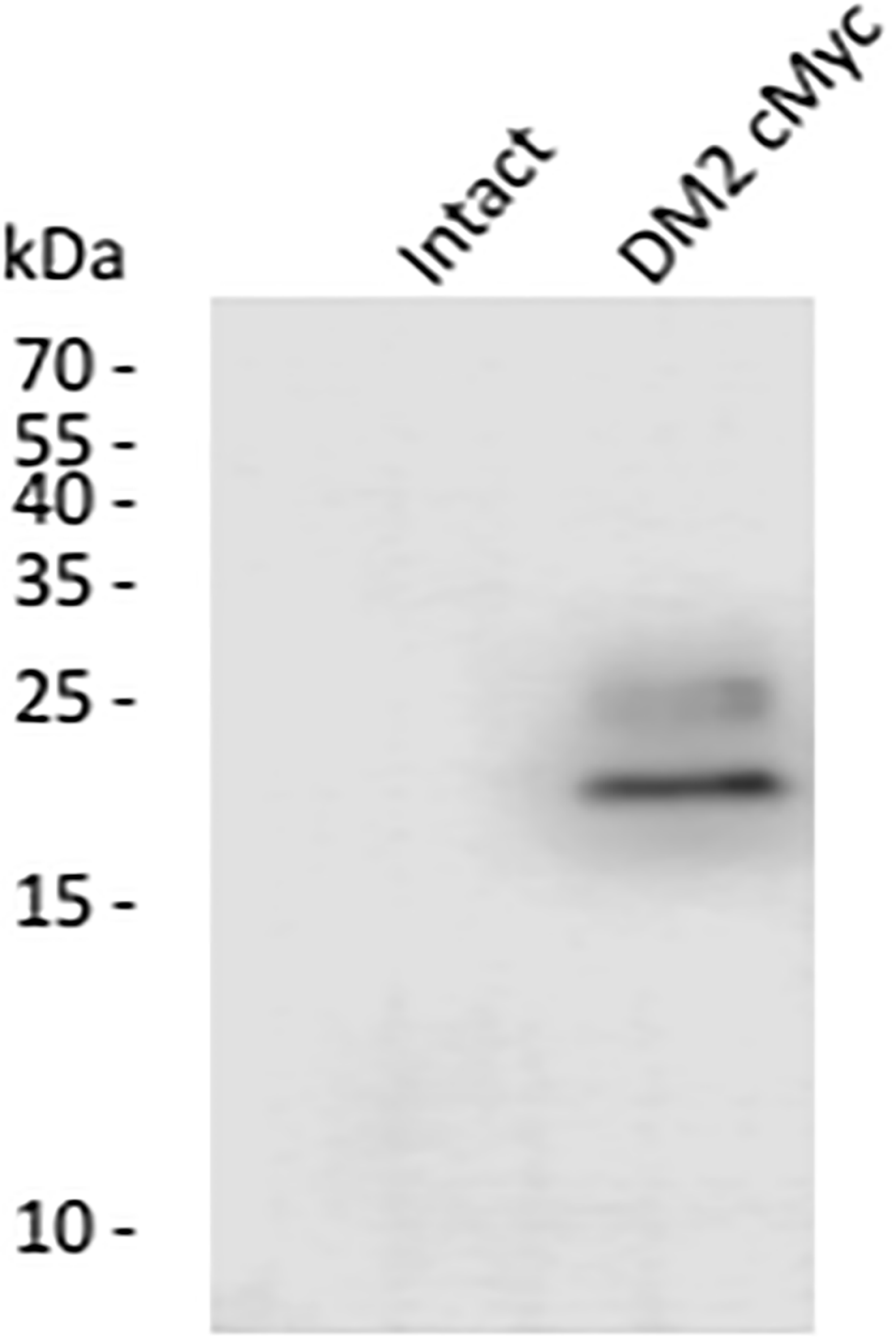
Western blot analysis. One each of the uninjected and the DM2 cMyc RNA injected oocyte was subjected to the Western Blot. The cMyc tag specific mouse monoclonal antibody 9E 10 was used to detect the expressed protein. The protein size is given in kDa.

To confirm the expression of DM2–cMyc protein, a tryptic mass spectrometry [33] was performed on oocytes incubated for 2 days after injection. A peptide of 36-amino acid long was identified, Arg123—Lys158, which included the carboxyl terminal of DM2, the spacer, and a portion of the cMyc-tag (Figs 1 and 4; S1 Table). In addition, the expression of DM2-cMyc protein was confirmed by Western blot analysis (Fig 5). Uninjected and DM2-cMyc RNA injected oocytes were used, and unique bands were found in the DM2-cMyc sample. The estimated molecular weights of the lower and upper bands were 20 and 23 kDa, respectively. The lower band corresponded to the predicted mass of the DM2-cMyc protein, 18304. A potential reason for the additional mass in this experiment was glycosylation at Asn39 (Fig 1). Modeling predicted this part of the polypeptide to be on the outside of the oocyte, because Asn39 was located in the leading amino terminus of the transmembrane helix in the model (Fig 2). When 6 oocytes were used in the preparation, stronger multiple bands were observed in the DM2-cMyc sample between 25 and 75 kDa. Aggregation, glycosylation and proteolysis may have been the cause of the multiple bands.

DM2 was considered to have been sorted into the plasma membrane, as in the case for the AM2 and the CM2, whereas the carboxyl-terminus cMyc-tag was assumed to be in the cytosol. The fragment that we identified may have arisen because the DM2–cMyc protein was not protected from the membrane disruption due to the trypsin treatment. Nevertheless, the observation by microscopy was supported by the results obtained by the mass spectrometry and the Western blot.

### Ion channel activities for DM2 protein

**Fig 6:**
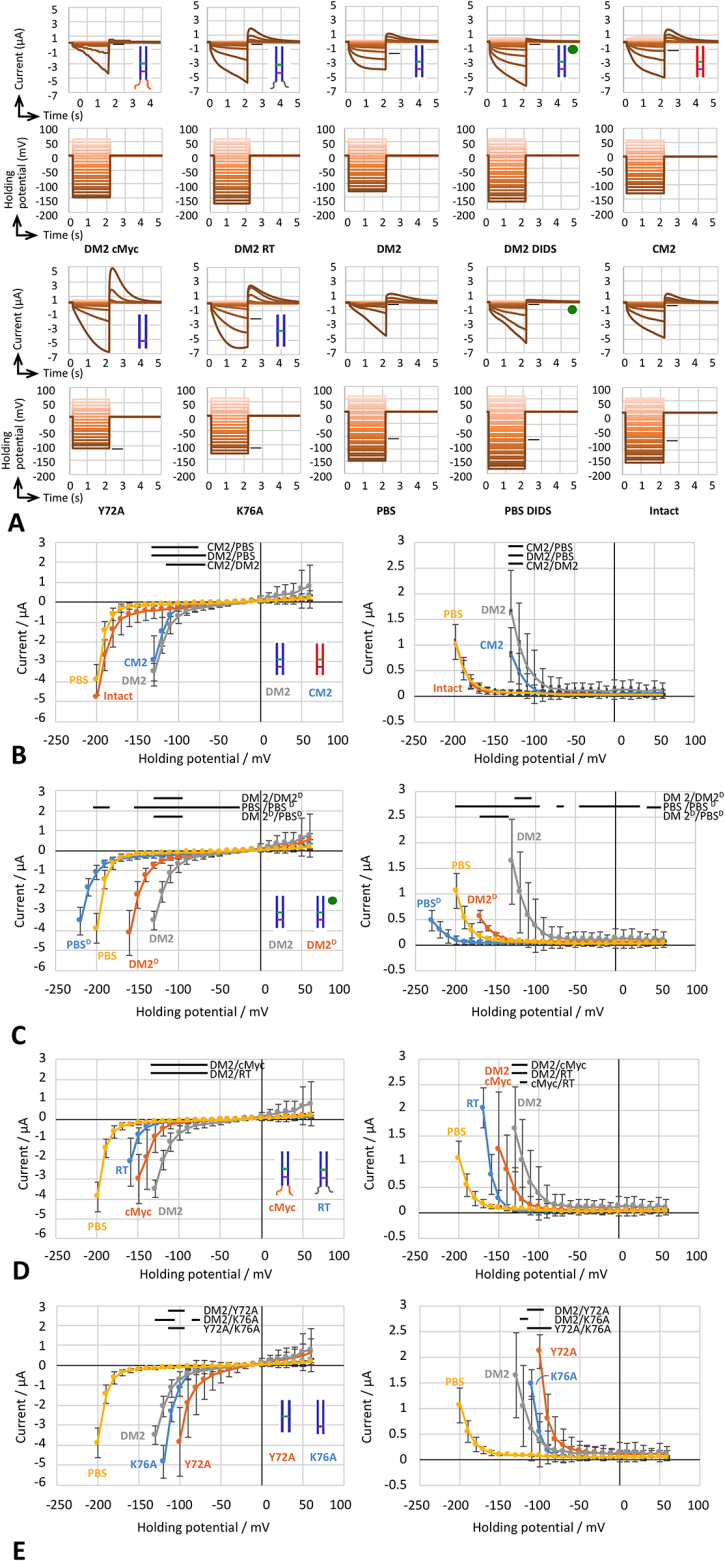
Induced current recorded by two-electrode voltage-clamp (TEVC) method at pH 8.5. A. Induced current and given membrane potential. An oocyte injected with either RNA or phosphate-buffered saline was subjected to a TEVC recording, and the current was measured for 2 s while the potential was kept constant at each of several membrane potentials with 10 mV intervals. A relaxation time of 5 s was set before the next measurement while the membrane was held at Vm = 0 mV. Short bars indicate values at -110 mV and the corresponding current for eye references. B-E. Overlapping traces for activating and tail current vs. holding potential. An average of 5 measurements were plotted with the SD. Solid bars indicate p<0.05 in the t-test. Cartoons represent the molecular organizations of M2 proteins. The green circle and D represents DIDS.

A defolliculated *Xenopus* oocyte and an oocyte injected with PBS were subjected to the TEVC method after 2 days of incubation (Fig 5). Both oocytes exhibited an inward current of approximately −5 μA when the membrane voltage was maintained at −200 mV (Fig 6A and 6B). The behaviors of intact and PBS-injected oocytes were similar, and the injection *per se* did not change the electrophysiological signature of the oocytes. The inward current most likely occurred via the innate voltage-gated chloride channel in the oocytes [34–37].

The oocyte injected with mRNA encoding DM2–cMyc and that injected with PBS were compared in terms of their electrophysiological behaviors using the TEVC method. The DM2–cMyc-expressing oocytes showed an inward current of −3.0 μA at a membrane voltage of −150 mV, whereas the PBS-injected oocyte showed an inward current of −0.2 μA (Fig 6A and 6D). Thus, we interpreted that the inward current observed in DM2–cMyc RNA-injected oocyte was due to the voltage-gated ion channel activity of the expressed protein. The charge—voltage Boltzmann distribution model was applied to represent the gating voltage as the midpoint of the sigmoid curve, V_mid_ (Table 1). The averaged tail V_mid_ values for DM2–cMyc-injected oocyte and PBS-injected oocyte were −149 and −206 mV, respectively. During the TEVC method, the DM2–cMyc-injected oocyte was more fragile than the PBS-injected oocyte, and we often failed to clamp the membrane voltage. Taking these findings together, we assumed that the same experimental procedure was applicable to other constructs, including DM2 without a tag.

**Table 1:** Activating and tail V_mid_ ^a^ values fitted by the charge–voltage Boltzmann distribution model (N = 5).

Oocyte	Activating V_mid_ (mV)	SD (mV)	Tail V_mid_ (mV)	SD (mV)
CM2	-141	± 10	-122	± 2
DM2	-139	± 3	-127	± 1
DM2 Y72A	-103	± 2	-109	± 1
DM2 K76A	-120	± 3	-129	± 1
DM2 cMyc	-156	± 11	-149	± 1
DM2 RT	-168	± 4	-171	± 1
PBS	-203	± 2	-206	± 2
DM2 DIDS ^b^	-162	± 3	-163	± 4
PBS DIDS ^b^	-226	± 2	-230	± 3

^a^ Mean simulated membrane potential that leaves half of ion channels open (V_mid_) by the charge—voltage Boltzmann distribution model. Correlation coefficient in the fitting of the Boltzmann curve was at least 0.90.

^b^ Administrated DIDS at 400 μM.

To test whether DM2 has ion channel activity, the TEVC method was used to observe the voltage-gated current in oocytes injected with CM2- or DM2-encoding RNA (Fig 6A and 6B). CM2 and DM2 mRNA-injected oocytes were also very fragile. Nevertheless, the CM2 construct used in this study provided an induced inward current of −2.9 μA when the membrane voltage was maintained at −130 mV (n = 5), which was consistent with the observation previously reported by Hongo *et al*. [25, 26]. The DM2 construct gave an inward induced current of −3.5 μA at a membrane voltage of −130 mV. The V_mid_ values for CM2- and DM2-injected oocytes were −122 and −127 mV, respectively.

To assess the ionic species passing through the DM2 ion channel, the reversal potential was measured using two consecutive recordings. The first run was to monitor ion channel activity and activation of the ion channels; the second was to estimate the reversal potentials. The reversal potentials for CM2 and DM2 were −22 and −21 mV, respectively (Table 2). The valence of the ionic species was consistent with a pure Cl^−^ permeability [38] when the internal and external concentrations of chloride ions were 40 mM [39] and 103.6 mM (ND96), respectively. When the sodium chloride in the ND96 solution was replaced by sodium methanesulfonic acid [26], reversal potentials were reduced to −7 and −6 mV for CM2 and DM2, respectively. For the DM2, the reversal potentials in reduced Na^+^ and increased K^+^ were -20 and -22 mV, respectively. To further assess the DM2 properties, a stilbene disulfonate chloride channel inhibitor, 4,4’-diisothiocyano-2,2’stilbenedisulfonic acid (DIDS), was used [40]. Administration of 400 μM of DIDS reduced both activating and tail current at the holding potential less than -110 mV (Fig 6A and 6C). The reversal potential of DM2 injected oocytes was reduced from -21 mV to -16 (± 1) mV. In the PBS injected oocytes, the reversal potentials were with and without DIDS, -21 (± 5)mV and -22 (± 5) mV, respectively. DM2 can be clarified as a DIDS-sensitive ion channel. Taking these findings together, we concluded that DM2 exhibited chloride ion channel activity under the same conditions in which the CM2 ion channel activity was reproduced [26].

**Table 2:** Reversal potential (RP; N = 5).

M2	RP in ND96 (mV)	RP in ND96 replaced Cl^−^ (mV) ^a^	RP in ND96 replaced Na^+^ (mV) ^b^	in ND96 increased K^+^ (mV) ^c^
DM2	−20.95 ± 1.05	−5.84 ± 0.40	−20.2 ± 0.31	−22.3 ± 7.1
DM2 Y72A	−21.77 ± 0.77	−9.75 ± 1.97	N/T	N/T
DM2 K76A	−21.19 ± 1.34	−14.03 ± 2.34	N/T	N/T
CM2	−22.05 ± 1.19	−6.52 ± 2.06	N/T	N/T

^a^ Reduced [Cl^−^] from 103.6 mM to 7.6 mM by replacing 96 mM NaCl in ND96 with 96 mM methane-sulfonic acid sodium salt.

^b^ Reduced [Na^+^] from 96.4 mM to 0.1 mM by 96 mM NaCl in ND96 with 96 mM N-methyl-D-glucamine hydrochloride.

^c^ Increased [K^+^] from 2 mM to 40 mM by using 40 mM KCl and by simultaneously reducing NaCl from 96 mM to 58 mM in ND96.

N/T, Not tested.

### Key amino acids for the gating in DM2 protein

We hypothesized that the YxxxK sequence motif is involved in ion channel function in DM2 (Fig 1A and 1B). To confirm the roles of the motif, we prepared two variants of the motif, AxxxK and YxxxA, by site-directed mutagenesis. The oocyte injected with mRNA of the mutated constructs was more stable than that injected with DM2. The observed gating voltages represented by tail V_mid_ for Y72A- and K76A-mutated DM2 were −109 and -129 mV, respectively (Table 1; Fig 6A and 6E). Y72A-DM2 showed activating V_mid_ values different from that of native DM2 (−127 mV). Mutated DM2, namely Y72A and K76A, showed similar reversal potentials of −22 and −21 mV, respectively (Table 2). However, the replacement of chloride ions in the ND96 solution caused the reversal potentials to differ between the two types of mutated DM2. This suggested that the YxxxK motif is involved in the gating function.

The DM2–cMyc-injected oocyte showed more negative V_mid_ than the native DM2-injected oocyte. Compared with native DM2, DM2–cMyc has extra 13 amino acids, including a spacer and cMyc-tag, at its C-terminal (Fig 1C). We hypothesized that this extended C-terminal affects the ion channel function. We constructed a DM2 variant with extra 16 amino acids at the carboxyl end of DM2 (DM2 RT) to mimic the cMyc-tag. The tail V_mid_ value for DM2–RT-injected oocyte was −171 mV, which was more negative than that for DM2–cMyc (Table 1; Fig 6A and 6D). Thus, additional amino acids at the carboxyl end probably alter channel function (Fig 1C).

## Discussion

The gating activity of M2 proteins involves amino acid side chains, namely through cation—pi interactions [41]. In the channel activation of AM2, lowering the pH gradually opens the Trp41 gate first, followed by a decrease in the deprotonation barrier of the His37 tetrad [21]. Trp41Ala substitution in AM2 was reported to result in the complete loss of ion channel activity [42]. In the case of CM2, which lacks Trp41 in AM2, Hongo *et al*. [25] reported a modest acid activation. A change of pH from 8.5 to 5.5 increases the relative current by approximately 1.3-fold in CM2. In DM2, we recorded the induced current at pH 8.5, 6.5, and 5.5. Nevertheless, there weren’t significant change in the induced current.

In the case of the AM2 helical bundle, the linkage of neutral imidazole rings of four His37 residues and the physical blockage by bulky indole rings of Trp41 facilitate the closing of the valve [43]. From protonation of the imidazole rings, the cation—pi interaction arises and the channel eventually opens. In the DM2 helical bundle, there are four potential cation—pi pairs, YxxxK. The average cation—pi interaction energy for the interacting pair Y-K was reported -4.1 kcal/mol [44]. Using this value, the sum of the interaction energy was: (−4.1) × 4 = −16.4 kcal/mol. When we set the internal concentration of chloride ions as 40 mM [39], the free energy change corresponding to Cl^−^ transfer from inside to outside the cell [45] can be calculated by the sum of the ion concentration gradient and membrane electrical potential at −152 mV: ΔGc = *RT* ln(40/103.6) = −0.4 kcal/mol; ΔGm = *FE* = 96,500 (C/mol) x 0.152 (V) x 1.99 (kcal) / 8.314 kJ = −3.5 kcal/mol; ΔG = ΔGc + ΔGm = (−0.4) + (−3.5) = −3.9 kcal/mol. The energy barrier by the YxxxK motif in terms of cation—pi interactions, −16.4 kcal/mol, is sufficiently large to regulate the ion flow, −3.9 kcal/mol.

The reversal potentials of the ion channels (Table 2) suggested that DM2, Y72A, and K76A conduct chloride ions as CM2 does, as their reversal potentials in ND96 solution (103.6 mM Cl^−^) do not differ significantly. Namely, the mutant reversal potentials in chloride-substituted ND96 (7.6 mM Cl^−^) are too different from those of CM2 and DM2 to suggest chloride specificity. Where, E_Cl_ = 58 (mV) x log(7.6 mM /40 mM) = +42 mV. These results suggested that the mutations interfere with the ion channel, but the specific mechanism involved in this is yet to be determined.

Nevertheless, the cation—pi interactions malfunction when either cation or pi-structure is missing. In the case of DM2, in the YxxxK motif, Tyr and Lys are potentially charged negatively and positively at pH 8.5, respectively. DM2 is likely to have a valve mechanism different from that of AM2 because Tyr and Lys can potentially form both ionic and cation—pi interactions [46, 47]. With DM2 Tyr72Ala, the pi provider was removed, so the amino acid remained hydrophobic. In DM2 Lys76Ala, the cation was removed and a hydrophobic side chain was introduced. Each step requires a certain amount of energy. In both types of mutated DM2, the gating potential was decreased, although this decrease was greater in Y72A than in K76A (Table 1). This suggested that Tyr72 provides a greater energy barrier than Lys76.

Although this report shows that DM2 functions as an ion channel, we continue to make efforts to clarify the molecular mechanism underlying the regulation of ion flow and to elucidate the functional evolutionary pathway of the four M2 proteins.

## Materials and methods

This research was performed under the supervision of the Institutional Biosafety Committee at the University of Nebraska—Lincoln (Protocol Number 174).

### Construction of plasmids and mRNA synthesis

A *Xenopus* oocyte expression vector, pNCB1 (GenBank accession number MF984401), was constructed based on pGEMHE [32], which contains a T7 promoter, β-globin 5′-UTR, multiple cloning sites, β-globin 3′-UTR, and a poly-A sequence. Native CM2 [Locus, YP_089658.1 (C/Ann Arbor/1/50)] and native and modified DM2 [Locus, AFJ19025 ((D/Swine/Oklahoma/1334/2011)] were synthesized by adding sequences corresponding to a restriction enzyme site immediately before and after the coding sequence and were cloned into pNCB1. *Bam*HI and *Xba*I were used for 5′ and 3′ ends, except in the case of CM2 (*Sma*I was used at the 5′ end). DNA was prepared by GenScript (Piscataway, NJ, USA). Before mRNA preparation, pNCB plasmid clones were linearized by *Hind*III and recovered by ethanol precipitation using half volume of 5 M ammonium acetate and two volumes of 100% ethanol. Invitrogen Ambion mMESSAGE mMACHINE T7 Transcription Kit (Thermo Fisher, Waltham, MA, USA) was used for mRNA synthesis. mRNA was recovered by LiCl precipitation and dissolved into PBS comprising 137 mM NaCl, 2.7 mM KCl, and 1.8 mM Na_2_HPO_4_. mRNA was stored in −20°C.

### Injection of mRNA-containing oocyte

Pipettes were pulled with a pipette puller, P-97 (Sutter Instrument, Novato, CA, USA), to set the tips at diameters of <22.5 μm and lengths of approximately 2.5 cm. Pipette tips were inspected with Microforge MF-830 (Narishige International USA, East Meadow, NY, USA) to ensure that they were smooth at the edges, so as not to tear the oocyte membrane, and to verify that the diameter was acceptable. Defolliculated *Xenopus* oocytes were purchased from *Xenopus* 1 (Dexter, MI, USA). Oocytes were injected using Auto-Nanoliter Injector Nanoject II (Drummond Scientific, Broomall, PA, USA). Each oocyte was placed in ND96 pH 8.5 buffer (96 mM NaCl, 2 mM KCl, 1.8 mM CaCl_2_, 1 mM MgCl_2_, and 5 mM HEPES-NaOH) [26] and injected with 50.6-nl mRNA. Other oocytes were injected with PBS to be considered as controls.

### Immunofluorescent imaging

MYC Mouse Antibody 9E 10 (Developmental Studies Hybridoma Bank, University of Iowa, Iowa City, IA, USA) was used to detect the cMyc-tag. Defolliculated oocytes injected with cRNA or PBS and incubated for 5 days were fixed in 4% paraformaldehyde and blocked in 5% horse serum in Tris-buffered saline (TBS) solution. The oocytes were incubated in 1:500 anti-myc mouse antibody 9E10 diluted in 1% skim milk in TBS-T solution (TBS containing 0.05% Tween-20) and were then incubated in 1:250 Alexa Fluor 488 donkey anti-mouse antibody (Thermo Fisher) diluted in 1% skim milk in TBS-T solution. Oocytes were then observed under the Olympus FV500 Confocal Microscope at the Morrison Microscopy Core Research Facility at the Center for Biotechnology in the University of Nebraska—Lincoln (CBT UNL).

### Mass spectrometry

An oocyte injected with RNA encoding cMyc-tagged DM2 was transferred to an Eppendorf tube with an aliquot of ND96 medium. After the removal of the ND96 medium, 2-μg trypsin in 200 μl of 50 mM NH_4_HCO_3_ in PBS (137 mM NaCl, 2.7 mM KCl, 10 mM Na_2_HPO_4_, and 10 mM KH_2_PO_4_) was added and was incubated at 25°C. After 5 h, 5 μl of solution was sampled and was run on a 0.075mm x 250mm C18 CSH column (Waters, Milford, MA, USA) by nano-liquid chromatography coupled to tandem mass spectrometry (nano-LC-MS/MS) [48], which is equipped with a Dionex RSLCnano U3000 and a Q-Exactive HF mass spectrometer (Thermo Fisher). This oocyte kept the globular outer shape for over 10 h, whereas the oocyte treated using trypsin with ND96 was disrupted in 10 min. Mascot (Matrix Science, London, UK; version 2.5.1) was used to analyze all the MS/MS samples. Mascot was set up to search the cRAP_20150130 (113 entries), the Uniprot of *Xenopus laevis* (June 2017, 42878 entries) databases and a custom database containing the DM2–cMyc sequence. Trypsin was the digestion enzyme. Mascot was searched with a fragment ion mass tolerance of 0.060 Da and a parent ion tolerance of 10.0 PPM. The deamidation of asparagine and glutamine and the oxidation of methionine were specified in Mascot as variable modifications. Scaffold (version Scaffold_4.8.1; Proteome Software Inc., Portland, OR, USA) was used to validate the MS/MS-based peptide and protein identifications. The identifications of peptide were accepted if they could be established at a 1% false-discovery rate (FDR) by the Peptide Prophet algorithm with Scaffold delta-mass correction [49]. This part of the work was performed by the Proteomics and Metabolomics Facility at CBT UNL.

### Western blot analysis

Western blot analysis. After 3 days of incubation in ND96, either intact or injected by DM2 cMyc RNA, oocytes were homogenized in 20 μL of HEPES buffer, pH 7.4, and centrifuged for 5 min at 800 x g at room temperature [50]. The proteins from 10 μL of each supernatant were separated by sodium dodecyl sulfate polyacrylamide gel (20%) electrophoresis. Proteins were transferred to a polyvinylidene fluoride membrane (0.2 μm pore size) that was blocked in Tris-buffered saline (TBS) containing 5% nonfat milk. The blot was stained with mouse anti-cMyc 9E10 in 2% BSA, washed with 0.1% Tween in TBS, and stained with a goat anti-mouse horse radish peroxidase conjugate in 5% nonfat milk in TBS. C-DiGit imager (LI-COR Biosciences, Lincoln, Nebraska, USA) was used to acquire the enhanced chemiluminescence signal.

### Electrophysiological recording

Glass electrodes (approximately 5 cm in length) were prepared by the P-97 puller. The tips were approximately 3 mm long and ≤22.5 μm in diameter. Each electrode was filled with 3 M filter-sterilized KCl and was placed on the voltage and current electrode holders of the Oocyte Clamp Amplifier OC-725C (Warner Instruments, Hamden, CT, USA). The OC-725C amplifier was connected to the Digidata 1550A Digitizer (Molecular Devices, Sunnyvale, CA, USA). Each oocyte was placed in a bath containing pH 8.5 ND96. The bath electrodes were set, and the voltage and current electrodes were used to puncture the oocyte. pCLAMP software (Molecular Devices) was used to control the holding potential of the oocyte and to record the resulting currents. The holding potential was decreased from 60 to −200 mV incrementally by −10 mV for 2 s at each step. The measurements of CM2 ended at −130 mV, of DM2 at −160 mV, and the measurements of control ended at −200 mV, depending on the capability of the clamping. The Boltzmann charge—voltage option in the pCLAMP program (Molecular Devices) was used to perform the data analysis [38, 51]. Where the Boltzmann Model is given: f (V) = I_max_ (1 + exp((V_mid_ − V)/V_C_) + C, where V, membrane potential; I_max_, maximal current; V_mid_, membrane potential when current is half-maximal; V_C_, voltage required to change I e-fold; C: constant y-offset. Curve fitting was performed using activating current at 2 s and tail current at 2.3 s. The membrane potential that resulted in half the maximal opening of the ion channels (V_mid_) was obtained as one of the representative values. The Nernst Potential Calculator was used to calculate the equilibrium potential (PhysiologyWeb at www.physiologyweb).

### Structural informatics

The prediction of transmembrane domains was performed using TMHMM [52]. The secondary structure was predicted using the Jpred server [53]. The isoelectric points and hydrophobicity represented by the GRAVY parameter were calculated using the ProtParam tool [54]. Prediction of glycosylation was performed using NetNGlyc 1.0 Server (www.cbs.dtu.dk/services/NetNGlyc/). The structural model for DM2 was compiled using the NMR structure of AM2 [23] as a template on the SWISS-MODEL server [55]. Graphics were prepared using the PyMOL Molecular Graphics System, Version 1.8 (Schrödinger, LLC, New York, NY, USA).

## Supporting information

S1 Table The fragmentation table for the corresponding the peptide Arg123-Lys158 representing both B- and Y-ions. (DOCX)
