# Supplementary material for: Influenza D virus M2 protein exhibits ion channel activity in Xenopus laevis oocytes
Source: PLoS One. 2018 Jun 21;13(6):e0199227. doi: 10.1371/journal.pone.0199227 (PMC6013169; doi:10.1371/journal.pone.0199227)
Supplement: S1 Table — (DOCX) [file pone.0199227.s001.docx]

S 1 Table. The fragmentation table for the corresponding the peptide Arg123-Lys158 representing both B- and Y-ions.

| B | B Ions | B+2H | B-NH3 | B-H2O | AA | Y Ions | Y+2H | Y-NH3 | Y-H2O | Y |
| --- | --- | --- | --- | --- | --- | --- | --- | --- | --- | --- |
| 1 | 157.11 | 79.06 | 140.08 |  | R | 3,765.72 | 1,883.36 | 3,748.69 | 3,747.71 | 36 |
| 2 | * 272.14 | 136.57 | 255.11 | 254.12 | D | 3,609.62 | 1,805.31 | 3,592.59 | 3,591.61 | 35 |
| 3 | 329.16 | 165.08 | 312.13 | 311.15 | G | 3,494.59 | 1,747.80 | 3,477.56 | 3,476.58 | 34 |
| 4 | * 458.20 | 229.60 | 441.17 | 440.19 | E | 3,437.57 | 1,719.29 | 3,420.54 | 3,419.56 | 33 |
| 5 | * 587.24 | 294.12 | 570.22 | 569.23 | E | 3,308.53 | 1,654.77 | 3,291.50 | 3,290.52 | 32 |
| 6 | * 688.29 | 344.65 | 671.26 | * 670.28 | T | 3,179.48 | * 1,590.25 | 3,162.46 | 3,161.47 | 31 |
| 7 | * 775.32 | 388.16 | 758.30 | 757.31 | S | 3,078.44 | 1,539.72 | 3,061.41 | 3,060.43 | 30 |
| 8 | * 862.35 | 431.68 | 845.33 | * 844.34 | S | 2,991.40 | 1,496.21 | 2,974.38 | 2,973.39 | 29 |
| 9 | * 959.41 | 480.21 | 942.38 | 941.40 | P | 2,904.37 | * 1,452.69 | 2,887.34 | 2,886.36 | 28 |
| 10 | * 1,088.45 | 544.73 | 1,071.42 | * 1,070.44 | E | 2,807.32 | 1,404.16 | 2,790.29 | 2,789.31 | 27 |
| 11 | * 1,217.49 | 609.25 | 1,200.47 | * 1,199.48 | E | 2,678.28 | 1,339.64 | 2,661.25 | 2,660.27 | 26 |
| 12 | * 1,274.51 | * 637.76 | 1,257.49 | 1,256.50 | G | 2,549.23 | 1,275.12 | 2,532.21 | 2,531.22 | 25 |
| 13 | * 1,387.60 | 694.30 | 1,370.57 | * 1,369.59 | L | 2,492.21 | 1,246.61 | 2,475.19 | 2,474.20 | 24 |
| 14 | * 1,444.62 | * 722.81 | 1,427.59 | * 1,426.61 | G | * 2,379.13 | * 1,190.07 | 2,362.10 | 2,361.12 | 23 |
| 15 | * 1,541.67 | * 771.34 | 1,524.65 | 1,523.66 | P | * 2,322.11 | * 1,161.56 | 2,305.08 | 2,304.10 | 22 |
| 16 | * 1,638.72 | 819.87 | 1,621.70 | 1,620.71 | P | * 2,225.05 | * 1,113.03 | * 2,208.03 | 2,207.04 | 21 |
| 17 | * 1,751.81 | 876.41 | 1,734.78 | * 1,733.80 | L | * 2,128.00 | 1,064.50 | 2,110.97 | 2,109.99 | 20 |
| 18 | * 1,838.84 | 919.92 | 1,821.81 | * 1,820.83 | S | * 2,014.92 | 1,007.96 | 1,997.89 | * 1,996.91 | 19 |
| 19 | * 1,895.86 | 948.43 | 1,878.84 | 1,877.85 | G | * 1,927.89 | 964.45 | 1,910.86 | * 1,909.87 | 18 |
| 20 | * 2,042.93 | 1,021.97 | 2,025.90 | 2,024.92 | F | * 1,870.86 | * 935.94 | 1,853.84 | 1,852.85 | 17 |
| 21 | 2,156.97 | 1,078.99 | 2,139.95 | 2,138.96 | N | * 1,723.80 | 862.40 | 1,706.77 | * 1,705.78 | 16 |
| 22 | 2,286.02 | * 1,143.51 | 2,268.99 | 2,268.01 | E | * 1,609.75 | 805.38 | 1,592.73 | * 1,591.74 | 15 |
| 23 | 2,400.06 | * 1,200.53 | 2,383.03 | 2,382.05 | N | * 1,480.71 | 740.86 | 1,463.68 | * 1,462.70 | 14 |
| 24 | 2,457.08 | * 1,229.04 | 2,440.05 | 2,439.07 | G | * 1,366.67 | 683.84 | 1,349.64 | 1,348.66 | 13 |
| 25 | 2,556.15 | 1,278.58 | 2,539.12 | 2,538.14 | V | * 1,309.65 | 655.33 | 1,292.62 | 1,291.64 | 12 |
| 26 | 2,703.22 | 1,352.11 | 2,686.19 | 2,685.21 | F | * 1,210.58 | 605.79 | 1,193.55 | * 1,192.57 | 11 |
| 27 | 2,834.26 | * 1,417.63 | 2,817.23 | 2,816.25 | M | * 1,063.51 | 532.26 | 1,046.48 | * 1,045.50 | 10 |
| 28 | 2,963.30 | 1,482.15 | 2,946.27 | 2,945.29 | E | * 932.47 | 466.74 | 915.44 | * 914.46 | 9 |
| 29 | 3,064.35 | 1,532.68 | 3,047.32 | 3,046.34 | T | * 803.43 | 402.22 | 786.40 | 785.42 | 8 |
| 30 | 3,177.43 | * 1,589.22 | 3,160.41 | 3,159.42 | L | * 702.38 | 351.69 | 685.35 | 684.37 | 7 |
| 31 | 3,234.45 | 1,617.73 | 3,217.43 | 3,216.44 | G | * 589.29 | 295.15 | 572.27 | * 571.28 | 6 |
| 32 | 3,305.49 | 1,653.25 | 3,288.46 | 3,287.48 | A | * 532.27 | 266.64 | 515.25 | 514.26 | 5 |
| 33 | 3,362.51 | 1,681.76 | 3,345.49 | 3,344.50 | G | * 461.24 | 231.12 | 444.21 | * 443.22 | 4 |
| 34 | 3,491.55 | 1,746.28 | 3,474.53 | 3,473.54 | E | 404.21 | 202.61 | 387.19 | * 386.20 | 3 |
| 35 | 3,619.61 | 1,810.31 | 3,602.59 | 3,601.60 | Q | * 275.17 | 138.09 | * 258.14 | 2 |  |
| 36 | 3,765.72 | 1,883.36 | 3,748.69 | 3,747.71 | K | 147.11 | 74.06 | 130.09 | 1 |  |

This table was exported by Scaffold (version Scaffold_4.8.1; Proteome Software Inc., Portland, OR, USA).

Asterisks (*) refer the ions were found. Colors were applied for the visibility.

The peptide sequence identified was RDGEETSSPEEGLGPPLSGFNENGVFMETLGAGEQK with the peptide identification probability

99.70%. Parameters obtained were: Previous amino acid, R; Next amino acid, L; Mascot Ion score, 116; Mascot Identity score, 29.7; Mascot Delta Ion score, 116; Actual peptide mass (AMU), 3,764.7; Spectrum charge, 3; Actual minus calculated peptide mass (AMU), 0.0055; Actual minus calculated peptide mass (PPM), 1.5; Peptide start index, 123; Peptide stop index, 158.
